# Diet and Drugs in Diabetes

**Published:** 1904-12-03

**Authors:** 


### DIET AND DRUGS IN DIABETES.

In an interesting article on the dietetic treatment of diabetes Dr. Robt. Hutchison [1] makes a number of useful and practical suggestions. Etiological considerations apart, it is sufficient for purposes of treatment to define diabetes as "a permanent glycosuria." For such a patient there is no routine diabetic diet, and every case requires individual study and consideration. The first necessary step is to determine the degree of tolerance for carbohydrates which the patient possesses. This is best done by means of a "test diet" containing a moderate amount of animal food, but excluding milk, and a known quantity of carbohydrate—such, for example, as is contained in four ounces of bread. After three days the total sugar excreted in 24 hours is estimated and compared with the 66 grammes of sugar represented by the carbohydrate in the permitted daily bread allowance. If the amount is less than 66 grammes there must obviously be some degree of tolerance of carbohydrates and the estimate of this may be made more exact by continuing the diet with a smaller allowance of bread. On the other hand, if the output of sugar exceeds 66 grammes there is no tolerance for carbohydrates and the case must be regarded as a severe one. The diet in these two groups requires different directions. In the former, that is in the comparatively mild cases, the patient may be allowed some carbohydrate, but always considerably less than the amount he is capable of assimilating. Say, for example, he can take four ounces of bread without any glycosuria, he may be allowed to have two ounces daily, and at occasional intervals even this should be withheld. In this way it may be possible gradually to increase the capacity for assimilating carbohydrates. On the other hand, if the test already described shows the case to be of the "severe" type, much caution is necessary. Here, as a first step, the urine should be tested for oxybutyric acid and its allies, which imply a greater or less danger of coma. This should be done by the addition of a few drops of a solution of perchloride of iron which, in the presence of the substances just mentioned, gives to the urine a dark port-wine colour. In face of such a result any change in diet must be made very gradually and the patient should be ordered sodium bicarbonate in doses of 4 to 8 drachms daily. Then, gradually, an attempt to reach a pure proteid and fat diet may be made. But in such a case it may be found that loss of weight or other sign of deterioration of the general health compels one to abandon all attempts to keep the urine sugar-free and makes it necessary to allow a definite quantity of carbohydrate in the form of bread and milk. On the contrary, if—though the case be shown by the preliminary test diet to be a severe one—the perchloride test is negative, one may either gradually or at once institute a strict diet, which means the exclusion of all carbohydrates. Regarding the value of individual foods to the diabetic, it may be said that fat stands in the front rank. Hence the patient should be encouraged to take such substances as butter, cream, bacon, and salad-oil; if there is difficulty in digesting fat, a little alcohol at meals is often useful. Animal foods are all free to the diabetic excepting only milk (which should be used in the sugar-free form), oysters, and liver, the last two containing some glycogen. Cheese has the advantage of being a food of animal origin with a large percentage of fat. Milk undoubtedly often increases both glycosuria and thirst and must be watched in these respects; a strict diet demands the sugar-free variety. Bread, except as already defined, must be banished from the dietary; the best substitute is the casoid-meal bread. Gluten bread is unpalatable and is never really free from starch. A common error is to suppose that to toast wheaten bread is to make it suitable for the diabetic. As a matter of fact toast is, weight for weight, richer in carbohydrate than untoasted bread. Potatoes have a distinct value in the dietary in appropriate cases; they contain less than half the percentage of starch present in bread and they form an admirable medium for the administration of fat in the shape of melted butter. Green vegetables though containing a little starch are permissible, and offer another opportunity for the ingestion of fat. Roots, tubers, dried fruits, are interdicted, but nuts are valuable chiefly because of their richness in fat. In regard to beverages alcohol is undoubtedly a food for diabetics, and may be ordered in the form of spirits, whilst the sweeter wines, malt liquors, and liqueurs are excluded. Tea and coffee are admissible, as is also cocoa, provided the pure powder be used, and not one diluted with starch. In diabetic coma skimmed milk is the best article of diet, given alone or mixed with Vichy water; with it full doses of sodium bicarbonate should be prescribed. Here also alcoholic stimulants should be freely used. Much proteid, it should be remembered, facilitates the production of the acid materials on the presence of which the development of coma appears to depend.

In elderly diabetics, as a rule, less strictness of diet is demanded. If no complications are present it may be sufficient to stop the use of sugar without interdicting starchy foods. If there is a tendency to obesity, an all-round reduction in the quantity of food consumed will have a favourable influence. These patients also need care in reference to alcohol, as sometimes the glycosuria may be traced to an excess in this direction. These rules will usually be sufficient to keep the excretion of sugar within reasonable limits.

Sir James Sawyer [2] writes in confirmation of Mossé's conclusions in regard to the value of the potato as a food for diabetics. The latter found that an allowance of, roughly, 2 to 3 lb. of potatoes daily brought, in 19 out of 20 cases, diminution of the glycosuria, relief of the thirst, and general improvement in the condition of the patient. The suggestion is that potatoes are superior to bread in the diabetic dietary, not merely because they contain a smaller proportion of starch, but because they include considerable quantities of potash salts. Sir James Sawyer, in order that the salts may be retained, insists on the potatoes being cooked by steaming "in their skins." He has also had bread, cakes, and biscuits made from a flour prepared from potatoes and bran, and he finds these very acceptable and useful in the construction of a dietary for diabetic patients. He publishes a formula for these various food preparations with his article.

Dr. R. T. Williamsen,[3] in speaking of the value of drugs in diabetes, believes that sodium salicylate and aspirin, given separately or combined, have some value in diminishing the sugar excretion in certain of the milder cases. In the more severe cases they have no beneficial effect. The salicylate —the pure natural sodium salt is the best preparation—should be given in doses of ten, increased to fifteen, grains thrice daily ; in some instances the dose may need to be repeated four or five times a day. Peppermint water is the most suitable vehicle for the salicylate. Aspirin may be ordered in similar doses, and it should be noted that soda-water or other alkali is apt to decompose the drug and thus to cause gastric disturbance. Some patients are found to do better with the one drug than with the other, and occasionally the best results follow the prescription of the salicylate during the daytime with a single dose of aspirin at night. In the severe forms of diabetes, where the urine gives a deep brownish-red colouration with perchloride of iron, bicarbonate of sodium in large doses is necessary ; it may be taken in soda-water or milk.

Another medicinal agent which was advanced a few years ago as valuable in the treatment of diabetes is eucalyptus.[4] The dried leaves, 10 grains to the ounce, were infused in water for half an hour, and six ounces of the infusion sweetened with saccharine were taken twice daily. In 15 out of 45 cases this treatment was followed by disappearance of the sugar and by the apparently complete cure of the patient. But as little or no confirmation of these results has been afforded it is probable that eucalyptus has followed many other "valuable remedies" into the therapeutic lumber-room.

Dr. Geo. S. Keith,[5] whose "Plea for a Simpler Diet" is well known, reports a case of diabetes in a man of 37 years, who, after some weeks of the orthodox diet, was suffering much from thirst, and was passing large quantities of urine. Dr. Keith advised considerable restriction of the nitrogenous food and the addition of a small quantity of bread to the dietary. The result was seen in reduction of the quantity of urine to between 40 and 50 ounces, with a sugar analysis of 0·32 per cent. Mr. F. A. Monckton[6] writes from New Zealand to support Dr. Keith's methods. After a special study of diabetes extending over 25 years he makes no restriction in regard to diet except to point out that in most cases some one article of food may disagree, and, if so, this has to be abandoned.

Without placing too much stress on these somewhat extreme doctrines, there is no doubt that wide recognition is at the present day being paid to the principle that harm rather than good may follow the prescription of too rigid a dietary in cases of diabetes. A routine practice of giving to any patient with glycosuria a list of foods which may be taken and of another with foods which are forbidden, together with a prescription for opium or codeia pills, is probably a degree worse than letting the patient severely alone. What is needed is a careful study of each individual case in reference to the amount of sugar passed, the weight of the patient, and the influence of the diet upon these factors. All these elements must be estimated at frequent intervals. Further, it must be remembered that the specific gravity of the urine and the total output of sugar may be considerably diminished, that many of the symptoms which depend upon the excessive glycosuria may disappear under a restricted diet, and yet the patient may remain in great danger of coma, as shown by a positive reaction of the urine to the perchloride of iron test. Hence, and more especially in the younger patients, a careful watch must be kept in this direction. It is well to add that opium and codeia are not remedies to be prescribed indiscriminately and as a matter of routine. Some years ago no less an authority than Dr. Dickinson[7] stated that he had practically given up the use of these drugs in the treatment of diabetes ; he found benefit rather in the prescription of nerve tonics and more especially of strychnine. In this connection one may note the advocacy of arsenic by Dr. Wm. Murray[8] of Newcastle-on-Tyne. His plan in cases of diabetes is to follow the usual rules of diet first and to order codeia. Then, having obtained the maximum benefit possible from this prescription, he orders arsenic. This he prefers in the form of the hydrochloric solution of which he gives 10 minims thrice daily, combining with the morning dose a little hydrochloric acid and strychnine. He maintains this treatment for at least three months, during which period he permits a gradual return to a saccharine and starchy diet. Some of his cases are undoubtedly very impressive and the treatment he advises is certainly worth a trial. But it is to be feared that in not a few cases of diabetes treatment proves of no avail to check the essential process of the disease and that all that can be hoped for is to ameliorate the condition of the patient and to check the rate of his downward movement.

[1] Practitioner, June 1904. [2] Brit. Med. Jour., March 5, 1904. [3] Lancet, Jan. 23, 1904. [4] Brit. Med. Jour., May 24, 1902. [5] Ibid., Feb. 5, 1902. [6] Ibid., May 17, 1902. [7] Lancet, Feb. 9, 1901. [8] Rough Notes on Remedies (H. K. Lewis, London).

